# Models and measurements of energy-dependent quenching

**DOI:** 10.1007/s11120-013-9857-7

**Published:** 2013-06-23

**Authors:** Julia Zaks, Kapil Amarnath, Emily J. Sylak-Glassman, Graham R. Fleming

**Affiliations:** 1 Physical Biosciences Division, Lawrence Berkeley National Laboratory, 1 Cyclotron Road, Berkeley, CA 94720 USA; 2 Graduate Group in Applied Science and Technology, University of California, Berkeley, CA 94720 USA; 3 Department of Chemistry, University of California at Berkeley, Berkeley, CA 94720 USA

**Keywords:** Non-photochemical quenching, Energy-dependent quenching, Fluorescence yield, Fluorescence lifetime

## Abstract

Energy-dependent quenching (qE) in photosystem II (PSII) is a pH-dependent response that enables plants to regulate light harvesting in response to rapid fluctuations in light intensity. In this review, we aim to provide a physical picture for understanding the interplay between the triggering of qE by a pH gradient across the thylakoid membrane and subsequent changes in PSII. We discuss how these changes alter the energy transfer network of chlorophyll in the grana membrane and allow it to switch between an unquenched and quenched state. Within this conceptual framework, we describe the biochemical and spectroscopic measurements and models that have been used to understand the mechanism of qE in plants with a focus on measurements of samples that perform qE in response to light. In addition, we address the outstanding questions and challenges in the field. One of the current challenges in gaining a full understanding of qE is the difficulty in simultaneously measuring both the photophysical mechanism of quenching and the physiological state of the thylakoid membrane. We suggest that new experimental and modeling efforts that can monitor the many processes that occur on multiple timescales and length scales will be important for elucidating the quantitative details of the mechanism of qE.

## Introduction

Oxygen-evolving photosynthetic organisms regulate light harvesting in photosystem II (PSII) in response to rapid changes in light intensity which occur during intermittent shading (Kulheim et al. 2002). Plants can, within seconds to minutes, turn on or off mechanisms that dissipate excess energy. The speed of these changes is faster than can be accounted for by changing gene expression, which can only take place within tens of minutes (Eberhard et al. 2008). From an engineering standpoint, the ability of a plant to dynamically regulate the behavior of the membrane without modifying its protein composition is particularly impressive. The design principles of this regulation would be useful as a blueprint for artificial photosynthetic systems such as solar cells and for engineering plants to optimize biomass or production of a natural product.

Energy is absorbed by chlorophyll in antenna proteins, which are transmembrane pigment–protein complexes in the thylakoid membrane (Blankenship 2002). The absorbed energy is then transferred to PSI and -II reaction centers (RCs) in the thylakoid membrane which convert the excitation energy to chemical energy through a charge separation event. Charge separation begins a chain of electron transport reactions that ultimately lead to the reduction of NADP^+^ to NADPH and to the production of ATP. When the rate of energy absorption exceeds the rate at which energy can be used in the electron transport chain, excited chlorophyll can no longer be efficiently quenched at the RC. The accumulation of excited chlorophyll (^1^Chl*) in PSII is dangerous to the plant. One major damage pathway is oxidative damage, which can occur when unquenched (^1^Chl*) undergoes intersystem crossing (ISC) to form triplet-state chlorophyll (^3^Chl) (Durrant et al. 1990). ^3^Chl reacts with ground state oxygen to generate ^1^O_2_, which can damage PSII (Barber 1994; Melis 1999). To reduce oxidative damage, plants have evolved mechanisms through which they are able to dissipate excess energy harmlessly. These mechanisms are collectively called non-photochemical quenching (NPQ) because the quenching does not result in the productive storage of energy.

There are NPQ mechanisms in all oxygen-evolving photosynthetic organisms, including cyanobacteria, algae, mosses, and plants (Niyogi and Truong 2013). Most of the work studying NPQ mechanisms has been done in plants. The mechanisms of NPQ in plants are generally broken down into energy-dependent quenching (qE), state transitions (qT) (Minagawa 2011), photoinhibition quenching (qI) (Müller et al. 2001), and zeaxanthin-dependent quenching (qZ) (Nilkens et al. 2010). Mechanisms are sometimes grouped by the timescales of activation and relaxation (Demmig-Adams and Winter 1988). Because the processes that give rise to NPQ are not fully understood, it is not clear whether the different components of NPQ involve entirely different mechanisms.

**Fig. 1:**
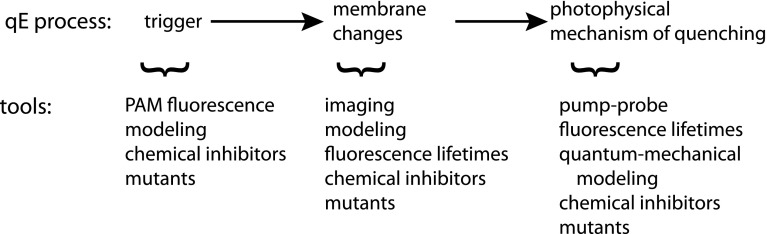
To understand the mechanism of qE requires an understanding of the dynamics of the trigger, the membrane change, and the photophysical mechanism. The techniques that are used to study the different aspects of the mechanism are listed below the respective process

**Fig. 2:**
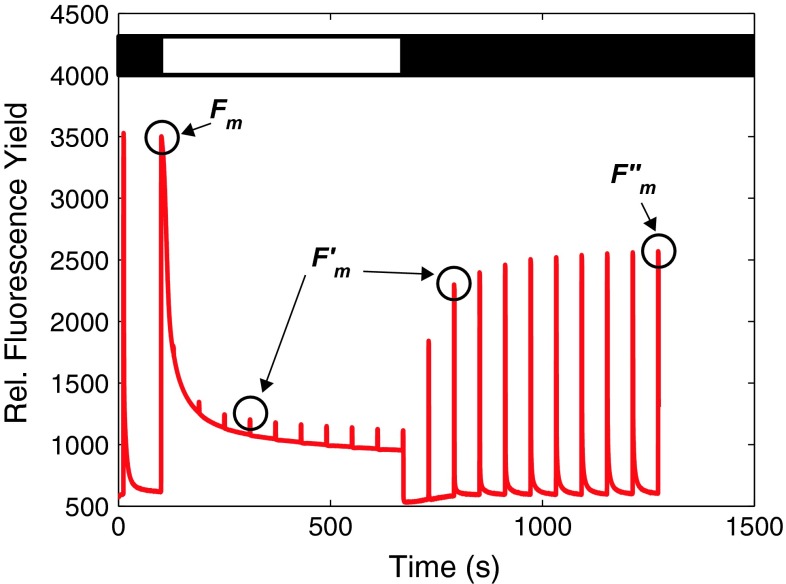
A PAM trace of a leaf from *Arabidopsis thaliana* is shown in *red*. The *bar* at the *top* of the figure indicates periods of darkness (*black*) and actinic light illumination at an intensity of 680 μmol photons m^−2^ s^−1^ (*white*). The saturating pulses occurred wherever there is a spike in fluorescence. The trace was averaged over six different leaves. The *F*_m_ *peak* and the $F_{\rm m}^{\prime\prime}$ *peaks* are *indicated*. The $F_{\rm m}^{\prime}$ *peaks* are all the peaks in fluorescence that are not *F*_m_ and $F_{\rm m}^{\prime\prime},$ and only two of them are pointed out for clarity

Efforts to understand qE have been underway for over 45 years, primarily on plants, but the mechanisms associated with qE are not fully known. In Fig. 1, we propose a definition of what it would mean to fully understand qE, inspired by Fig. 2 from Ruban’s 2012 review (Ruban et al. 2012). Firstly, it is necessary to understand the trigger or what conditions cause qE to turn on. While it is known that a pH gradient $(\Updelta\hbox{pH})$ across the thylakoid membrane triggers qE (Ruban et al. 2012), to fully understand the role of the pH trigger, it is necessary to characterize the modifications of pH-sensitive moieties. Secondly, it is important to understand the membrane changes that occur to create a qE-active state and how the properties of particular pigments are altered to be able to rapidly quench excitation. It is thought that a macroscopic membrane rearrangement may induce conformational changes in individual proteins that affect the interactions between pigments, changing the energy transfer dynamics (Betterle et al. 2009; Johnson and Ruban 2011). Lastly, it is crucial to understand the photophysical quenching mechanisms, where and how quenching occurs. The mechanism and the location of quenching have been under debate for many years. Quenching through chlorophyll–chlorophyll interactions (Beddard and Porter 1976; Miloslavina et al. 2008; Müller et al. 2010) and chlorophyll–carotenoid interactions (Ahn et al. 2008; Bode et al. 2009; Gilmore et al. 1995; Holt et al. 2005; Pascal et al. 2005; Ruban et al. 2007) have been proposed. qE has been studied by researchers from a broad range of fields. This diversity of approaches has led to a wide variety of theoretical and experimental tools that have been valuable in studying qE.

In this paper, we review the methods and techniques that have been used in qE research. These methods, though often developed and primarily used to study plants, can be used to study qE in any photosynthetic organism, and many can be used to study any NPQ mechanism. We focus on the applications of these methods to samples that are capable of performing qE in response to light, such as thylakoids, chloroplasts, and whole leaves, and do not review many experiments done on isolated and aggregated proteins. For a review of experiments on isolated complexes, see Ruban et al. (2012). We also limit the scope of this review to the application of these methods to qE in plants, although other organisms, such as cyanobacteria, also exhibit NPQ processes that have similarities with qE. Some methods, such as the use of fluorescence yield measurements, chemical inhibitors, and qE mutants, have been used to extract information about all parts of the qE process: the trigger, membrane change, and photophysical mechanism of quenching. We discuss the use of these methods, as well as their strengths and limitations, in the “General tools for the study of qE” section. In the “Triggering of qE” section, we discuss the current understanding of the trigger by reviewing methods and models for correlating qE with the lumen pH. We discuss the techniques used to monitor membrane changes and to identify the quenching site(s) and photophysical mechanism(s) of NPQ in the “Formation of qE in the grana membrane” section. Finally, in the “New tools for characterizing qE in vivo” section, we discuss the development of measurements and techniques to study the dynamics of qE in vivo.

## General tools for the study of qE

### Discovery and early studies of qE

**Fig. 3:**
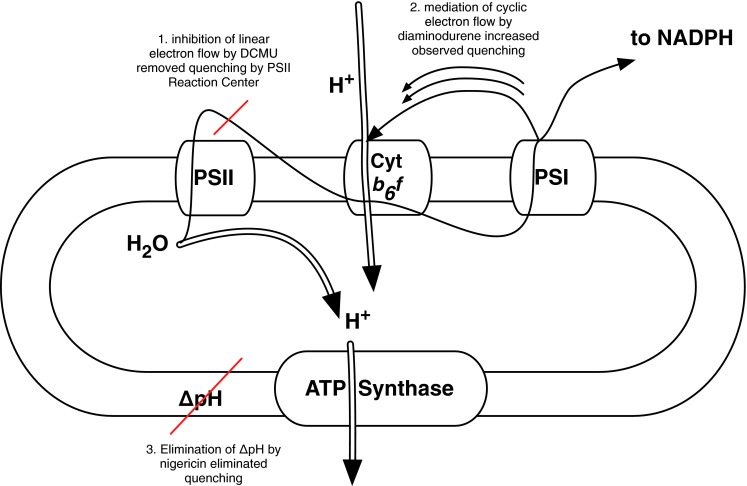
Schematic of experiment performed by Wraight and Crofts (1970) to identify that the $\Updelta\hbox{pH}$ was the trigger for qE. The *thin black arrows* indicate electron flow and the *thick arrows* with the *white stems* refer to proton movement. In the experiment, chloroplasts were treated with DCMU to prevent quenching by the PSII reaction center. The addition of diaminodurene to these chloroplasts lowered the lumen pH via cyclic electron flow and caused chlorophyll fluorescence to be quenched. This quenching was eliminated by the addition of nigericin and dianemycin, which dissipate the pH gradient. The quenching was much less sensitive to the addition of valinomycin, which dissipates the electric field across the membrane

qE was first observed in fluorescence studies of isolated chloroplasts subjected to chemical treatments. The amount of chlorophyll fluorescence was found to depend on the pH of the lumen. Figure 3 illustrates the series of experiments performed by Murata and Sugahara (1969) and Wraight and Crofts (1970). Chloroplasts were first treated with dichlorophenyl-dimethylurea (DCMU), which inhibits electron transfer at PSII and prevents photochemical quenching. Because excited chlorophyll could not be quenched photochemically (by charge separation at the RC), a high level of fluorescence was observed. The addition of mediators of cyclic electron flow (either phenazine methosulfate [PMS] Murata and Sugahara 1969 or diaminodurene [DAD] Wraight and Crofts 1970), which stimulate the formation of a $\Updelta\hbox{pH}$ across the thylakoid membrane, quenched the fluorescence. This quenching was eliminated by the addition of ionophores that dissipated the $\Updelta\hbox{pH},$ but was not eliminated by dissipation of the electric field gradient $\Updelta \psi.$ These experiments led to the observation that this “energy-dependent quenching,” now abbreviated as qE, is triggered by the $\Updelta\hbox{pH}$ across the thylakoid membrane. Nearly a decade after these initial studies of a pH-dependent quenching mechanism, Briantais et al. (1979) found that this phenomenon was not something that could only be seen under artificial treatments, but occurs naturally when plants are illuminated. Briantais and coworkers correlated the chlorophyll fluorescence with the pH of the lumen by measuring the pH-dependent fluorescence of 9-aminoacridine. They found that illuminated chloroplasts’ fluorescence yield decreases as the pH decreases. This result indicated that qE occurs naturally and not just with chemical treatments. The use of chemicals to block linear electron transport and uncouple the pH and electric field gradients is still a useful technique for studying qE.

### Fluorescence yield measurements

Chlorophyll fluorescence yield is the most frequently used quantity for observing qE. Because the chlorophyll fluorescence yield depends on the rates of relaxation for excited state chlorophyll, it can be used to determine the amount of photochemical quenching and NPQ (Krause and Weis 1991). Additionally, the fluorescence yield can be detected non-invasively, which has allowed researchers to measure the fluorescence yield in living photosynthetic organisms such as green algae and leaves as they respond to changing light conditions both in the laboratory and in the field.

Early fluorescence measurements (Murata and Sugahara 1969; Wraight and Crofts 1970) detected the absolute fluorescence from an illuminated sample and how it changed following different chemical treatments. Because the total fluorescence is proportional to the illumination intensity, comparing the amount of fluorescence across different illumination conditions requires measuring of the fluorescence quantum yield, $\phi_{\rm F}.$

1 $$ \phi_{\rm F} = \frac{\hbox{number of photons emitted}}{\hbox{number of photons absorbed}}. $$

PAM fluorimetry is a widely used tool for measuring changes in the chlorophyll fluorescence yield as plants acclimate to changing light conditions (Schreiber et al. 1986). PAM techniques are reviewed in Brooks and Niyogi (2011) and Schreiber (2004). While absolute fluorescence measurements use a single light source to illuminate the sample and induce fluorescence, PAM fluorimeters only detect fluorescence resulting from a low intensity (<0.1 μmol photons m^−2^ s^−1^) modulated measuring light that minimally affects the photochemistry or NPQ in the plant.

Typical qE PAM fluorimeter measurements consist of a dark-acclimated sample exposed to actinic light (light that results in productive photosynthesis) until qE reaches a steady state (approximately 10 min), followed by a period of dark reacclimation until qE turns off. To distinguish the effects of photochemical quenching (irreversible charge separation in the RC) and NPQ, fluorescence yield measurements are compared when PSII RCs are open and closed. RCs are considered to be open when the primary plastoquinone electron acceptor in the RC, *Q*_A_, is oxidized and is considered closed when *Q*_A_ is reduced (Baker 2008; Govindjee 2004). During the illumination and dark periods, short (<1 s) pulses of high intensity (up to 20,000 μmol photons m^−2^ s^−1^) actinic light are used to close PSII RCs. When RCs are open, excited chlorophyll can relax via photochemical quenching, NPQ, fluorescence, or ISC. When the saturating pulses close the RCs, the only available pathways are NPQ, fluorescence, or ISC. The rates of these processes affect the measured fluorescence quantum yield.

To characterize the NPQ response of a plant, it is useful to compare the fluorescence yield when the PSII RCs are closed before and during light acclimation. *F*_m_ is proportional to the maximum fluorescence yield measured during a saturating pulse of actinic light applied to dark-acclimated leaves. $F_{\rm m}^{\prime}$ is the maximum fluorescence yield following exposure to light, also measured during saturating pulses. A parameter called NPQ can be calculated with these parameters (Schreiber et al. 1994).

2 $$ \hbox{NPQ} = \frac{F_{\rm m}-F_{\rm m}^{\prime}}{F_{\rm m}^{\prime}}. $$

This NPQ parameter is useful to compare relative amounts of quenching between various mutants and light conditions because it increases as quenching turns on and decreases as quenching turns off. The derivation and use of this NPQ parameter are described in greater detail in the Appendix A and in Ahn et al.(2009), Baker (2008), Brooks and Niyogi (2011), and Holzwarth et al. (2013). To separate qE from qT, qZ, and qI, $F_{\rm m}^{\prime\prime},$ the maximum fluorescence yield after qE has relaxed, is often measured (Ahn et al. 2009; Johnson and Ruban 2011) and used instead of $F_{\rm m}^{\prime}$ in Eq. 2.

PAM traces also allow researchers to quickly assay the qE response with different mutants, light conditions, and chemical treatments. These measurements are often correlated with biochemical measurements that quantify parameters such as the protein or pigment content (for example, Betterle et al. 2009; Nilkens et al. 2010; Niyogi et al. 1998) to investigate the relationship between these components and qE.

### Chemical inhibitors

Chemical inhibitors have been used in in vitro measurements to perturb a plant’s qE response, often by inhibiting particular steps of photosynthetic electron transport (see Table 1). DCMU is commonly used to close RCs (Murata and Sugahara 1969) by blocking the electron flow from PSII to plastoquinone pool, effectively closing the RCs without using saturating light, as is done in PAM fluorimetry (Clayton et al. 1972). In this way, DCMU allows researchers to take measurements without photochemical quenching present. This allows for the isolation of NPQ processes without the complications of photochemical processes.

**Table 1:** Chemical treatments used to study qE

Names	Effects
*N*,*N*′-dicyclohexylcarbodiimide (DCCD)	Binds to protonatable protein carboxylate groups (Ruban et al. 1992)
3-(3,4-Dichlorophenyl)-1,1-dimethylurea (DCMU)	Blocks electron flow from PSII to plastoquinone, closes PSII reaction centers (Murata and Sugahara 1969)
Nigericin	Eliminates $\Updelta\hbox{pH}$ (Heldt et al. 1973)
Carbonylcyanide *m*-chlorophenylhydrazone (DCCP)	Dissipates $\Updelta\hbox{pH}$ and $\Updelta \varPsi$ (Nishio and Whitmarsh 1993)
Dithiothreitol (DTT)	Inhibits violaxanthin de-epoxidase (Yamamoto and Kamite 1972)
Gramicidin	Eliminates $\Updelta\hbox{pH}$ and $\Updelta \varPsi$ (Nishio and Whitmarsh 1993)
Dibromothymoquinone (DBMIB)	Blocks electron flow from plastoquinone to cytochrome *b*_6_ *f* (Nishio and Whitmarsh 1993)
Methyl viologen	Electron acceptor (Nishio and Whitmarsh 1993)
Diaminodurene (DAD)	Mediator of cyclic electron flow (Wraight and Crofts 1970)
Phenazine methosulfate (PMS)	Mediator of cyclic electron flow (Murata and Sugahara 1969)
Valinomycin	Eliminates $\Updelta \varPsi$ (Wraight and Crofts 1970)

Ionophores are used in qE studies to alter the $\Updelta\hbox{pH}$ and/or $\Updelta \psi.$ Nigericin is a commonly used chemical inhibitor in qE studies (Heldt et al. 1973). Nigericin is a proton-potassium anti-porter that dissipates the $\Updelta\hbox{pH}$ across the thylakoid membrane and eliminates qE. In both in vitro and in vivo studies, it has been particularly useful because it can be added while qE is already activated to dissipate the $\Updelta\hbox{pH}$ (Amarnath et al. 2012; Johnson and Ruban 2010). The addition of nigericin separates qE from the other NPQ components. There are other chemicals that can be used to alter the electrochemical gradient. Gramicidin and carbonylcyanide *m*-chlorophenylhydrazone (CCCP) dissipate both $\Updelta\hbox{pH}$ and $\Updelta \psi$ (Nishio and Whitmarsh 1993). Valinomycin, a potassium transporter, dissipates only the $\Updelta \psi$ (Wraight and Crofts 1970). These treatments were used to determine that the $\Updelta\hbox{pH},$ not the $\Updelta\psi,$ is the trigger for qE, as described in the introduction of this Section.

*N*,*N*′-dicyclohexylcarbodiimide (DCCD) binds to protonatable carboxylate groups accessible to the lumen in the hydrophobic region of proteins (Ruban et al. 1992). It has been used to determine whether a protein is pH sensitive and to identify protonatable residues in antenna complexes of PSII (Walters et al. 1996) and the protein PsbS (Dominici et al. 2002; Li et al. 2002b).

The enhancement of cyclic electron flow around PSI by chemical electron donors and acceptors such as PMS and DAD led to the discovery of qE, as discussed in the introduction of this section. This approach has been used to provide information about the trigger of qE because it enables researchers to manipulate the pH of the lumen without involving PSII. As an example, DAD has been used to decrease the pH of the lumen below physiological levels to investigate qE in mutants of *Arabidopsis thaliana* (Johnson and Ruban 2011).

More generally, a challenge in using chemical inhibitors is that they may have multiple interactions in the chloroplast that are not fully known or characterized. As a result, pathways other than the desired one may be affected.

### qE mutants

Plant mutants that display enhanced or inhibited quenching have aided in identifying the components that are necessary to see a full qE response. Many of these mutants were created by randomly mutating *A. thaliana* seeds by fast neutron bombardment, treatment with ethylmethyl sulfinate (EMS), or transfer DNA. Seedlings are selected and characterized by their fluorescence yield, often using a video imaging technique developed by Niyogi et al. (1998) that allows for rapid visualization of NPQ on a large number of mutagenized seedlings. Plants with altered NPQ levels compared to wild type can then be further characterized. This method allowed for the identification of many qE mutants. These mutants are listed in Table 2.

**Table 2:** *A. thaliana* mutants used to study qE

Names	Mutations	Effects
*npq4* (Li et al. 2000)	Lacks PsbS function	Decreased amount of qE, slower turn on and off compared to wild type
*npq1* (Niyogi et al. 1998)	No violaxanthin de-epoxidase activity	Decreased qE, slower turn on and off compared to wild type
*npq2* (Niyogi et al. 1998)	No zeaxanthin epoxidase activity	Equal qE, more rapid turn on, slower turn off compared to wild type
*lut2* (Pogson et al. 1998)	No production of lutein	Decreased amount of qE
*npq1lut2* (Niyogi et al. 2001)	See above	No qE
*npq4npq1lut2* (Li et al. 2002a)	See above	No qE
L5 (Li et al. 2002a)	Over-expresses PsbS	Increased amount of qE
L17 (Li et al. 2002a)	Over-expresses PsbS	Increased amount of qE
*npq4-E122Q* (Li et al. 2002b)	One of two lumen-exposed glutamate residues mutated to glutamine	50 % qE compared to wild type
*npq4-E226Q* (Li et al. 2002b)	One of two lumen-exposed glutamate residues mutated to glutamine	50 % qE compared to wild type

*Arabidopsis thaliana* mutants have provided researchers with a method of removing or altering proteins in the photosynthetic apparatus. Examples include the mutants which showed that the protein PsbS is necessary for qE. In wild type plants grown in low light, there are approximately 2 PsbS per PSII (Funk et al. 1995). The *npq4* mutant, which lacks PsbS, shows no qE in PAM traces, demonstrating that PsbS is necessary for qE in vivo (Li et al. 2000). The *npq4-E122Q* and *npq4-E226Q* mutants, each of which has one lumen-exposed glutamate residue mutated such that it cannot be protonated, have qE levels that are midway between that of the wild type and *npq4*. This showed that PsbS is pH sensitive and likely undergoes some conformational change when the lumen pH is low (Li et al. 2002b). To further examine the role of PsbS, the *npq4-1* mutant was complemented with the wild type *PsbS* gene, yielding a set of mutants with varying levels of PsbS (Niyogi et al. 2005). The qE levels of these mutants show that the maximum qE level increases with increasing ratio of PsbS to PSII (Niyogi et al. 2005). This increase eventually plateaus when the level of PsbS is 6–8 times that of the wild type. Additionally, two mutants that contain elevated levels of PsbS, L5 and L17, exhibit approximately twice the amount of NPQ compared to wild type plants. These mutants have revealed that the capacity for qE in wild type *A. thaliana* is not saturated and can be increased by elevating PsbS levels.

Because of the complexity and interconnectedness of the thylakoid membrane, removing one component, such as a pigment or a protein, may cause other components in the membrane to compensate in a manner that is challenging to predict and characterize. One example of this is the mutant *npq1*, which cannot convert violaxanthin to zeaxanthin (Niyogi et al. 1998). However, the mutation does not block the biosynthesis of zeaxanthin from β-carotene. Therefore, while *npq1* has a strongly reduced amount of zeaxanthin, some zeaxanthin and antheraxanthin are still present. In the case of *npq2*, which lacks zeaxanthin epoxidase, zeaxanthin accumulates even in the dark, so quenching components related to qZ are always present in the *npq2* mutant. This additional quenching is reflected in the lower *F*_v_/*F*_m_ quantity in *npq2* that is used to characterize the plant’s capacity for photochemistry and limits the qE-specific information that can be extracted from studies of the *npq2* mutant.

Another caution in using mutants is that changing one gene may have unintended consequences on the greater photosynthetic apparatus. For instance, knocking out PsbS as in *npq4* could change the properties of the thylakoid membrane, which affect more processes than just qE. PsbS has been shown to affect the stacking of the grana membranes (Kiss et al. 2008) and to affect the distance between PSII centers upon illumination (Betterle et al. 2009). These changes have not been shown to be directly related to qE, but they complicate the interpretation of the role of PsbS. As another example, the altered qE dynamics of the *lut2* mutant, which lacks lutein, may be due to the misfolding of light-harvesting proteins rather than a change in the qE mechanism (Dall’Osto et al. 2006). Nonetheless, the *A. thaliana* qE mutants have provided a powerful tool for studying the components and mechanism of qE.

## Triggering of qE

We now turn to a description of tools to study qE triggering. A complete understanding of the triggering of qE by $\Updelta\hbox{pH}$ requires characterizing the value of the lumen pH at which the components of qE are turned on. It is important to know the pH level at which any pH-sensitive qE components are activated and whether these pH levels are absolute or modulated by other environmental factors. It is also important to characterize the “steepness” of the pH dependence of qE. A steep pH dependence would correlate to a “switch” from fully on to fully off in a short pH range. By contrast, a shallow pH dependence would correspond to a “dial,” where the activation level gradually changes from off to on. In addition to quantifying the response of the proteins involved in qE to protonation, a complete understanding of qE triggering requires knowing the response of PSII to the protonation of these key proteins. This response could involve conformational changes within or between proteins and is discussed in the “Formation of qE in the grana membrane” section.

Although work with chemical inhibitors has convincingly shown that qE is triggered by acidification of the lumen, quantifying the qE response to lumen pH is challenging. This challenge arises from the fact that the complexes involved in qE are embedded in the thylakoid membrane and that the pH-sensitive components of these complexes are located in the lumen space. To characterize the response of qE to $\Updelta\hbox{pH},$ researchers have sought to measure the lumen pH and determine the p*K*_a_s of key proteins and enzymes. These downstream responses to the pH trigger have been investigated by a combination of measuring the lumen pH and correlating it to the amount of qE. The effect of $\Updelta\hbox{pH}$ on qE has been quantified by fitting the relationship between observed qE quenching and measured lumen pH to various equations, as in Takizawa et al. (2007), Johnson and Ruban (2011), and Johnson et al. (2012). Several experimental methods have been developed to measure the lumen pH as well as the $\Updelta\hbox{pH}$ across the thylakoid membrane. These methods rely on indirect spectroscopic measurements of lumen pH, either by measuring fluorescence of dyes (Junge et al. 1979; Schuldiner et al. 1972) or by measuring spectroscopic signals of carotenoids (Bailleul et al. 2010; Takizawa et al. 2007). In this section, we review several recent experiments investigating the triggering of qE.

### Proteins triggered by $\Updelta\hbox{pH}$

**Fig. 4:**
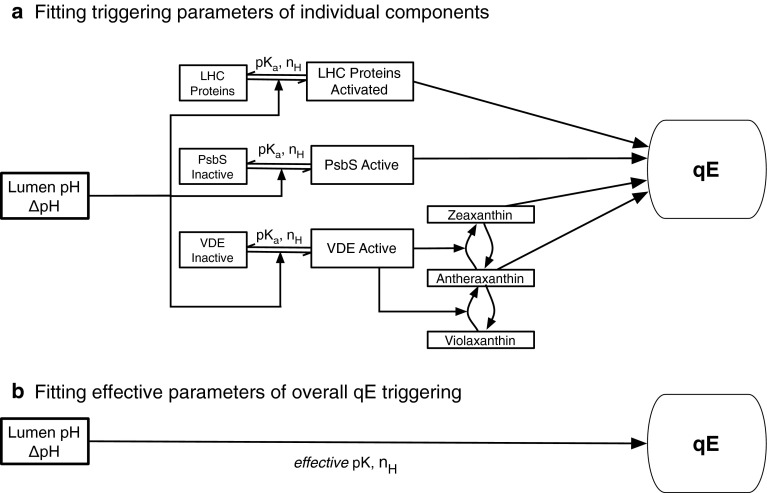
**a** The triggering of qE in plants by lumen pH involves the protonation of PsbS, VDE, and possibly other light-harvesting proteins. A full understanding of qE triggering involves quantitative knowledge of the p*K*_a_ and Hill coefficient of each protonation step, as well as a characterization of the interaction between pigments and protonated proteins to form a qE state. **b** Because activation levels of individual proteins cannot be measured directly, experimental data quantifying the relationship between qE to lumen pH frequently fit the overall data phenomenologically to an effective p*K*_a_ and Hill coefficient

Figure 4a illustrates the known components of qE in plants that respond to lumen pH. When the pH of the lumen drops and $\Updelta\hbox{pH}$ is formed across the membrane, several processes in the thylakoid membrane are triggered:

1. The enzyme violaxanthin de-epoxidase (VDE) is activated (Jahns et al. 2009). In its active form, VDE converts the carotenoid violaxanthin, which is present in several of the light-harvesting proteins of PSII, to the carotenoid zeaxanthin via the xanthophyll cycle.
2. The protein PsbS (Funk et al. 1995), which is necessary for rapidly reversible quenching in vivo, is activated (Li et al. 2000). The sensing of lumen pH is done by two lumen-exposed glutamates, as discussed in the “qE mutants” section.
3. The minor light-harvesting pigment–protein complexes CPs29 and -26 contain glutamate residues that bind DCCD (Walters et al. 1996). It is possible that the protonation of these residues contributes to triggering qE. Deletion of either light-harvesting complex (LHC) from the PSII antenna (Andersson et al. 2001; Betterle et al. 2009; de Bianchi et al. 2008) does not eliminate qE, suggesting that these complexes could play an indirect role in qE (Ruban et al. 2012). Nonetheless, qE turns on more slowly and reaches lower levels in mutants lacking CP29 (Betterle et al. 2009; de Bianchi et al. 2011).

Because the individual activation steps giving rise to qE cannot be measured directly, efforts to understand the relationship between lumen pH and the components of qE have largely relied on measurements of total qE, as illustrated in Fig. 4. We review these measurements below. In general, to quantify the relationship between lumen pH and qE, measurements have been fit to the Hill equation. The Hill equation can be used to describe the protonation of individual proteins, as in Fig. 4a. It is also commonly used as a more general phenomenological equation to fit data and has been directly applied to quantify the relationship between lumen pH and qE, as in Fig. 4b.

The Hill equation has the form

3 $$ F = \frac{[H^+]^n} {[H^+]^n +[10^{-p{\it K}_a}]^n}, $$

where *F* is the fraction of proteins that are activated. The Hill equation contains two parameters: the p*K*_a_, which is the pH at which *F* = 0.5, and the Hill coefficient *n*, which is a measure of the sigmoidicity, or “steepness,” of the transition of *F* from a “100 % on” state to a “100 % off” state. In the case when a protein must bind multiple protons to be activated, and when this binding is highly cooperative, the Hill coefficient *n* can be interpreted as the number of protons needed to activate the protein, as in the reaction

4 $$ A + n { H}^+ \rightleftharpoons A{ H}^+_n. $$

In the case when binding is not extremely cooperative, the Hill coefficient still measures the cooperativity of binding, but does not correspond directly to a physical property such as the number of protonatable sites (Weiss 1997).

The existing measurements from several labs fit quite well to the Hill equation. However, the Hill equation does not directly correspond to a physical model in most situations (Weiss 1997). As a result, extracting mechanistic information from measurements of qE measured as a function of lumen pH is challenging. One way forward is through the development of physically motivated mathematical models that explicitly incorporate each protonation event in various hypotheses of qE mechanism. In the following sections, we review measurements correlating lumen pH and the hypotheses that have been generated from these measurements.

### Measurements of qE triggering

#### ΔpH or low lumen pH?

For understanding the processes triggering qE, it is important to differentiate between those processes that only require a low lumen pH and processes that require a $\Updelta\hbox{pH}$ across the thylakoid membrane. The protonation of residues in PsbS, VDE, and LHC proteins can be accomplished by lowering the lumen pH, without necessarily requiring a pH gradient across the thylakoid membrane. However, work by Goss et al. (2008) demonstrated that a pH gradient across the thylakoid membrane, along with a neutral or slightly basic stromal pH, is required for the formation of zeaxanthin-dependent qE. Once qE is formed, it is possible to maintain qE even in the absence of a pH gradient if the lumen pH is kept sufficiently low (Rees et al. 1992). This property was used to determine the qE versus pH curves in Johnson and Ruban (2011) and Johnson et al. (2012). The ability to maintain qE in low pH, even without a $\Updelta\hbox{pH},$ suggests that the $\Updelta\hbox{pH}$ is required for proper insertion of zeaxanthin (Goss et al. 2008), but that other pH-sensitive components of qE do not require a pH gradient.

#### Correlation of qE with in vivo measurements of lumen pH

To quantify the response of qE to lumen pH in vivo, the Kramer group has measured the relationship between steady-state qE and lumen pH. The lumen pH was measured spectroscopically through a measurement of the electrochromic shift (ECS), which is a signal arising from the Stark effect of the electric field across the thylakoid membrane on the energy levels of carotenoids embedded in the membrane (Bailleul et al. 2010; Witt 1979). This effect causes the absorption spectrum of carotenoids in the spectral region between 450 and 550 nm to shift. The extent of spectral shift is proportional to the amplitude of the electric field and as a result can be used to measure the transmembrane electric field. The ECS measurement can be used to probe the lumen pH by shuttering off the actinic light and measuring the “reverse ECS.” Explanations of information that can be obtained from the ECS measurement, including measurements of the lumen pH, are given in Bailleul et al. (2010), Cruz et al. (2001), and Takizawa et al. (2007).

To estimate the p*K*_a_s of PsbS and of qZ in vivo, Takizawa and coworkers assumed that de-epoxidized xanthophyll (i.e., zeaxanthin or antheraxanthin) and protonated PsbS are the two components necessary for qE. This assumption involved fitting to a specific mechanistic model (Fig. 4a) and excluded the possibility that the protonation of LHC proteins is a factor in qE activation in vivo. Nonetheless, because it followed a specific model, this assumption enabled estimates of the pH level at which qE components were activated. The p*K*_a_ of PsbS activation was fitted to be 6.8, with a Hill coefficient of ∼1, and the effective p*K*_a_ of qZ was fit to be 6.8 with a Hill coefficient of 4.3. This effort is one of the first attempts thus far to fit the activation levels of qE using in vivo measurements, and the results suggest that the p*K*_a_s of PsbS and qZ are higher in vivo than the p*K*_a_s for isolated glutamate (Li et al. 2002b) and for VDE in vitro (Jahns et al. 2009).

Because of the challenges of estimating the lumen pH in vivo, the p*K*_a_ values reported will surely be subject to refinement and reexamination. Nonetheless, the spectroscopic approach of estimating p*K*_a_s and Hill coefficients is notable because the parameters are estimated from intact leaves. The approach of spectroscopically measuring the lumen pH through the ECS shift is unique and powerful in that it does not require the extraction of chloroplasts or the use of chemicals. The technique of using reverse ECS would be even more powerful it if could be extended to measure lumen pH over the course of light adaptation. Such a measurement could be used to fit mechanistic kinetic models of the protonation of the proteins involved in qE. Doing so would provide a method for determining the p*K*_a_ of qE components during the process of qE induction and would enable greater precision than steady-state measurements in measuring the p*K*_a_s and Hill coefficients of qE triggering.

### Titration of qE versus lumen pH

Chemical treatments and mutants in *A. thaliana* have shown that PsbS (Li et al. 2000), zeaxanthin (Demmig-Adams 1990; Niyogi et al. 1997), and lutein (Pogson et al. 1998) are responsible for the majority of qE in vivo. However, recent results from the Ruban group have suggested that qE-type quenching can be induced in the absence of any of these components by artificially lowering the lumen pH by mediating cyclic electron flow (Johnson and Ruban 2011; Johnson et al. 2012). Chloroplasts isolated from *npq4* and *npq1lut2* mutants of *A. thaliana* were able to quench chlorophyll fluorescence when the lumen pH in the chloroplasts was lowered below levels typically found in vivo. This quenching had many of the same properties of that from wild type chloroplasts, which led to the suggestion that PsbS and zeaxanthin modulate the p*K* of qE in the thylakoid membrane. These observations were extensions of earlier studies correlating qE and $\Updelta$pH in wild type *A. thaliana* (Briantais et al. 1979).

To characterize the effect of PsbS and zeaxanthin on the p*K* of qE, a titration of qE against lumen pH was performed (Johnson and Ruban 2011; Johnson et al. 2012). The $\Updelta\hbox{pH}$ was measured with 9-aminoacridine, and qE was fit to the equation

5 $$ \hbox{qE} = \hbox{qE}_{{\rm max}} \frac{\Updelta \hbox{pH}^n}{\Updelta \hbox{pH}^n + \Updelta\hbox{pH}_0^n}, $$

where *n* is the Hill coefficient and $\Updelta\hbox{pH}_0$ (p*K*) is the pH at which half of all protonatable residues are protonated. By assuming a stromal pH of 8.0, Johnson and coworkers extracted p*K*s and Hill coefficients for qE in the presence and absence of lutein and zeaxanthin. In this approach, the p*K* of qE was fit to a value of 4.2 in violaxanthin-bound *npq4*, and increased to a value of 6.3 in zeaxanthin-bound wild type. This approach, in which no assumptions are made about the interaction between the pH-sensing components of qE, is illustrated in Fig. 4b. The extracted p*K* and Hill coefficient are phenomenological parameters that serve to quantify qE triggering and are useful for comparing different mutants and chemical treatments. The maximum capacity for qE, qE_max_, was found to be 85 % of the wild type value in the *npq4* and *lut2npq1* mutants. Because this capacity was relatively high, Johnson and coworkers formulated the hypothesis that the role of PsbS, zeaxanthin, and lutein is to elevate the p*K* of qE, but that the photophysical process responsible for qE quenching could in principle proceed in the absence of these components at very low pH values. In this hypothesis, zeaxanthin and lutein have indirect roles in qE and are not the pigments involved in the dissipation of excitation energy (Johnson and Ruban 2011; Johnson et al. 2012; Ruban et al. 2012).

The advantage of studying qE in isolated chloroplasts using chemical treatments is that the chloroplast is a sufficiently intact system that qE can still be observed, yet the aqueous spaces within the chloroplast are accessible enough to the experimentalist that certain processes such as cyclic electron flow can be inhibited or up-regulated. Because these treatments shift the lumen pH far from the physiological conditions in which qE is normally observed, the hypotheses of qE mechanism formed on the basis of these studies must be subject to testing in vivo. One approach would be to construct quantitative predictions of hypotheses that are based on and inspired by the in vitro results and integrate those quantitative predictions into mathematical models that predict experiments such as PAM that can be non-invasively observed in a living system, as we describe in the “New tools for characterizing qE in vivo” section.

## Formation of qE in the grana membrane

**Fig. 5:**
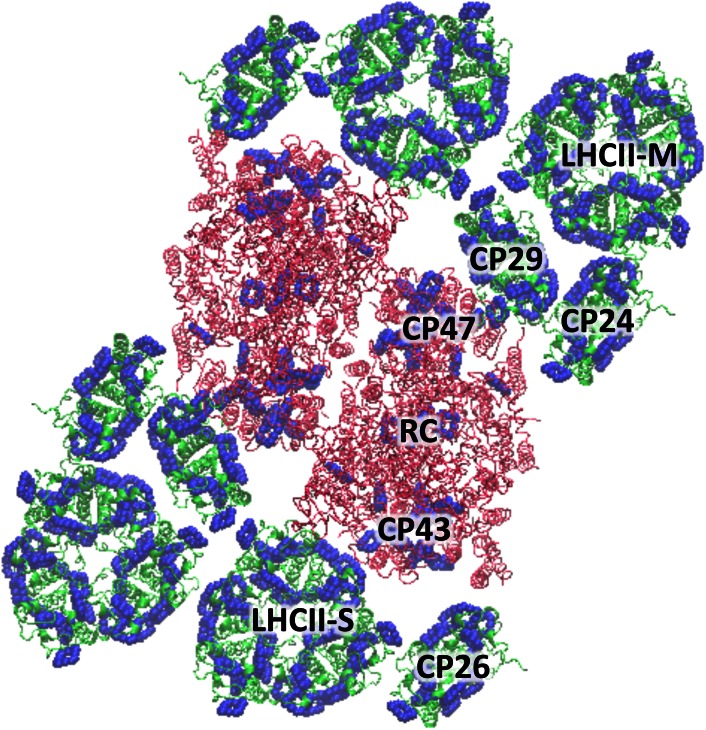
Structure of the PSII supercomplex, based on the recent electron microscopy images taken by Caffarri et al. (2009). The proteins are shown as *ribbons* and the light-absorbing chlorin part of the chlorophyll pigments are outlined by the *blue spheres*. The light-harvesting antenna proteins on the exterior of the supercomplex are *green*, while the reaction center core (CPs47, -43, and the RC, which consists of the D1 and D2 proteins) is *red*. The supercomplex is a dimer. S stands for strongly bound and M for medium-bound LHCIIs. The supercomplex is a dimer; one of the monomers is labelled

The protonation of the pH-sensitive proteins in the grana membrane triggers changes in PSII that turn on qE. A physical picture that captures those changes requires an understanding of how the organization of PSII and its antenna in the grana gives rise to its light-harvesting and quenching functionality (Dekker and Boekma 2005). The grana membrane is densely populated by PSII supercomplexes and major LHCIIs. LHCII is a pigment–protein complex that can reversibly bind to the exterior of PSII supercomplexes, which are composed of several pigment–protein complexes (Fig. 5). LHCIIs are located on the periphery, and RCs are located in the interior of PSII supercomplexes. Between the LHCIIs and RCs are the aforementioned minor LHCs, CPs24, -26, and -29. Together, the LHCIIs and PSII supercomplexes form a variably fluid array of proteins (Kouřil et al. 2012b). This array gives rise to an energy transfer network in which the pigments in the light-harvesting proteins absorb light and transfer the resulting excitation energy to RCs, where it is converted into chemical energy. In order to turn on chlorophyll quenching, this energy transfer network must change.

**Fig. 6:**
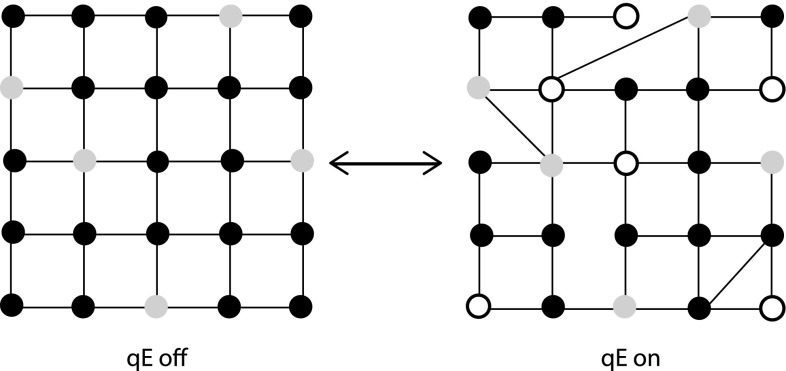
A schematic of a possible configuration of chlorophyll connectivity of a portion of the grana membrane when qE is off (*left*) and when qE is on (*right*). The *black circles* represent non-quenching chlorophyll, such as those in LHCII. The *gray circles* represent PSII reaction centers, and the *white circles* represent qE quenching sites. At both reaction centers and qE sites, there is a rate for removing excitation from the *grid*. The *grid lines* display the connectivity for energy transfer between different groups of chlorophyll

We represent the energy transfer network of the grana membrane using a simple grid in Fig. 6. We use this picture to illustrate the changes in the energy transfer network that may occur when qE turns on. It is a simplification and reduction of the complete network, which contains ∼100,000 chlorophylls and the description of which has not yet been conclusively determined (Croce and van Amerongen 2011). The nodes (circles) represent groups of chlorophylls at which excitation energy can be localized and are either antenna or RCs. The dark-acclimated membrane without qE is shown on the left. Excitation energy can be absorbed at any nodes and transferred on the picosecond (10^−12^s) timescale along the lattice grid lines until it reaches a RC (gray nodes) (van Amerongen et al. 2000). Once it reaches a RC, the excitation energy can be “photochemically” quenched and converted into chemical energy. The $\Updelta\hbox{pH}$ triggers a series of changes in the membrane (Fig. 6, right) that change the energy transfer network on a timescale of tens of seconds to minutes. Some antennae (Havaux et al. 2007) (white nodes) gain a photophysical pathway or mechanism with a rate of relaxation to the ground state that is fast relative to fluorescence and ISC. Efficient quenching of chlorophyll excitations could prevent the excitation from reaching a RC that is susceptible to damage. To alter the properties of the pigments such that they become quenching sites may require a rearrangement of the proteins in the membrane, which is indicated by the changes in the connectivity of the network.

While this general picture of quenching is agreed on, nearly all of the details remain controversial. The energetic connectivity of pigments in the membrane is determined by their orientation, separation from other pigments, and their local protein environments. However, it is not possible at present to acquire the nearly atomic level resolution necessary for obtaining that information. Instead, a few approaches are used to study intact photosynthetic organisms. We categorize these approaches into four groups: spectroscopic measurements of pigment–pigment interactions, imaging and microscopy, fluorescence lifetimes, and transient absorption (TA) spectroscopy. Combined with modeling, these techniques can provide insight on aspects of both the membrane changes and on the site and mechanism of qE (Fig. 1).

### Spectroscopic measurements of pigment–pigment interactions

To switch a pigment from participating in light harvesting (black node in Fig. 6) to quenching (white node) requires an alteration of its physical properties by changing its protein environment or by interactions with other pigments. Pigment–pigment interactions can be tuned by small changes in the protein conformation (van Oort et al. 2011) or by changes in the structure of a neighboring pigment, as when zeaxanthin replaces violaxanthin in high light (Crimi et al. 2001). This suggests that characterizing pigment–pigment interactions might identify pigments which are involved in qE and how those interactions might change to allow quenching to occur.

There are several methods to measure pigment–pigment interactions that are correlated with qE. One spectroscopic change that occurs during qE is the $\Updelta A_{535}$ signal (Krause 1973). This signal is determined by measuring the difference absorption spectra between light- and dark-acclimated leaves. At 535 nm, there is an increase in absorption, which, on the basis of quantum mechanical modeling, is thought to be due to interactions between two carotenoids (violaxanthin or zeaxanthin) that occur only under qE conditions (Duffy et al. 2010). Another indicator of qE is the change in the resonance Raman spectrum of the leaf around 953 cm^−1^ after 5 min of exposure to actinic light (Robert 2009; Ruban et al. 2007). This change is thought to be due to changes in the conformation of a neoxanthin carotenoid in LHCII. A third indicator of qE is an increase in far-red fluorescence thought to be emitted from LHCII (Johnson et al. 2011; Melis 1999). The $\Updelta A_{535}$ signal, the 953 cm^−1^ resonance Raman signal, and the fluorescence red shift have been observed in vitro under conditions that promote the aggregation of LHCII. Based partly on this evidence, Ruban and coworkers proposed that qE occurs due to the aggregation of LHCII in the membrane, which causes the formation of a qE quenching site (Ruban et al. 2007). Recently, the Walla group developed a method for measuring the coupling between carotenoid and chlorophyll S_1_ excited states and showed that this coupling increases during qE and correlates with qE (Bode et al. 2009; Wilk et al. 2013). Based on a proposal by van Amerongen and van Grondelle, these results were suggested to be due to an excitonic state formed between the S_1_ state of a carotenoid and the *Q*_y_ excited state of chlorophyll *a* that could quickly dissipate excitation energy (Bode et al. 2009; van Amerongen et al. 2001).

### Imaging and microscopy

Assessing the extent to which membrane rearrangement plays a role in qE requires tools that can probe the spatial arrangement of proteins in the grana membrane. Lower resolution images of the membrane that can resolve the PSIIs and LHCIIs are beneficial in determining whether a large rearrangement occurs and dramatically changes the energetic connectivity between chlorophylls. A rearrangement could be required for the conformational changes that switch a pigment into a quencher, or it could itself serve to disconnect LHCs from RCs. Protein dynamics in living systems is typically observed by tagging proteins with fluorophores. However, because most of the proteins of interest are integral membrane proteins and the grana membrane is up to 80 % protein (Kirchhoff et al. 2008a), such tagging is experimentally difficult. Additionally, the tightly stacked grana membrane prevents the introduction of fluorescent proteins within the stacked region because the proteins are large compared to the space between membranes. Moreover, there are few fluorescent proteins or dyes the excitation wavelengths of which do not coincide with those of carotenoids and chlorophylls. Because the resolution limit of optical microscopy is ∼200 nm, and due to the difficulties in tagging proteins of interest, protein organization in the thylakoid membrane cannot be currently resolved through confocal optical microscopy. As a result, electron microscopy (EM) and atomic force microscopy (AFM), which are more invasive than optical microscopy and can resolve features on a short length scale, have been used to image the thylakoid membrane (Dekker and Boekma 2005; Kirchhoff et al. 2008b).

EM imaging of *A. thaliana* has recently been used to understand the arrangement of proteins in the thylakoid (Boekma et al. 2000; Dekker and Boekma 2005; Kouřil et al. 2012a). Thylakoid membranes are isolated and then negatively stained for contrast. Betterle and coworkers observed that the distance between PSII centers decreased during acclimation in wild type *A. thaliana* but not in the *npq*4 mutant (Betterle et al. 2009). Another common EM technique is freeze-fracture EM, in which thylakoids are frozen and then split along the lipid bilayer such that the transmembrane proteins remain on one side of the split membrane (for review, see Staehelin 2003). Using freeze-fracture EM, the Ruban group observed clustering of the LHCs on the timescales of qE induction (Johnson et al. 2011). One drawback of using these EM techniques is the intensive sample preparation that is required. Negative staining requires fixing and dehydrating the grana, and freeze-fracture images are made with metallic replicas made from the frozen samples. In this way, the sample preparation techniques may impact the arrangement of proteins (Kirchhoff et al. 2008b). To cope with these experimental drawbacks, there has recently been effort to use cryo EM and tomography to image unstained spinach and pea chloroplasts. In cryo EM, thylakoids or chloroplasts are flash frozen at cryogenic temperatures to create vitreous samples that can then be sectioned (Dall’Osto et al. 2006; Kouřil et al. 2011). The advantage to cryo EM is that the samples remain hydrated, with the water in the sample forming a non-crystalline, vitreous ice. This technique has allowed Kouřil to examine the native 3D structure of the grana membrane and the arrangement of PSII within the membrane (Kouřil et al. 2012b). Although there are some experimental challenges associated with cryo EM (Daum et al. 2010; De Carlo et al. 2002), it shows much promise for future use in studying the organization of proteins in the chloroplast before and during qE.

In addition to EM-based techniques, researchers have imaged thylakoid membranes using AFM. In AFM, samples are placed on a mica surface exposed to air and probed with a cantilever. An image is created using the height of the sample for contrast (Kirchhoff et al. 2008b). One drawback of this technique is that having the sample exposed to air rather than immersed in liquid may affect membrane properties (Fukuma et al. 2007). The application of new water-based AFM techniques (Liu et al. 2011) could probe the native rearrangements that take place in the thylakoid. Such imaging techniques should be extremely valuable for assessing the changes in chlorophyll connectivity in the membrane. In addition, thermodynamic models will be useful for understanding the strength and directionality of energetic interactions between proteins required for causing changes in membrane organization (Drepper et al. 1993; Kirchhoff et al. 2004; Schneider and Geissler 2013). It will be important to use images and models of membrane rearrangements to interpret fluorescence lifetimes, a technique that is discussed in the next section.

### Fluorescence lifetimes

**Fig. 7:**
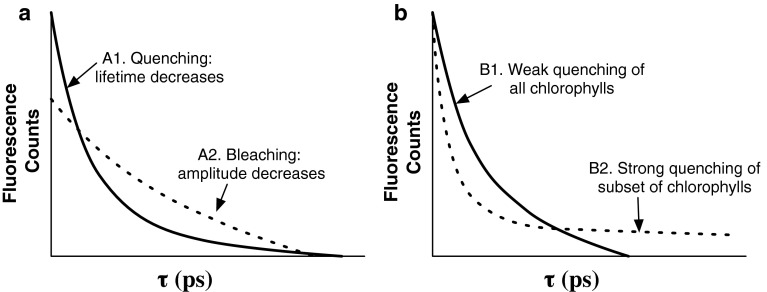
Scenarios that give rise to indistinguishable fluorescence yield measurements, but that can be distinguished by fluorescence lifetime measurements. **a** Illustration of fluorescence lifetimes of quenching (*case A1*, *solid line*), which reduces the fluorescence lifetime, and bleaching (*case A2*, *dashed line*), which reduces the overall fluorescence amplitude. These two situations could give the same fluorescence yields even thought they display different fluorescence lifetimes. **b** Illustration of fluorescence lifetimes of moderate quenching of all fluorophores (*case B1*, *solid line*) and strong quenching of a small fraction of fluorophores (*case B2*, *dashed line*) which cannot be differentiated using fluorescence yield measurements

The chlorophyll fluorescence lifetime measures the relaxation of the chlorophyll excited state and contains information about the energy transfer network of the grana membrane. The benefits of lifetime measurements can be seen in scenarios that give rise to the same fluorescence yield, but different fluorescence lifetimes. Figure 7a illustrates the difference between quenching (A1), in which the lifetime of the excited state is shortened, and bleaching (A2), in which the number of fluorophores decreases. Because the fluorescence yield, which is measured in the PAM experiment, is equal to the area under the fluorescence lifetime curve, PAM measurements cannot differentiate between bleaching and quenching. Figure 7b illustrates how two different energy transfer networks can be resolved by measuring fluorescence lifetimes, but not by measuring fluorescence yields.

The two decays in Fig. 7b correspond to two different energy transfer networks. For instance, the fast component of B2 could be due to chlorophylls that are very close to sites with high quenching rates and the slow component due to chlorophylls far from quenching sites. The excited state lifetime is affected by any properties that affect the energy transfer network, including the location of the quenchers with respect to the light harvesters, the connectivity between chlorophylls, and the rate of quenching at qE sites. Extracting this information requires the use of models of energy transfer and trapping. Interpretation of fluorescence lifetime data is dependent on the sample preparation and on the energy transfer models used to analyze the data.

The methods for measuring fluorescence lifetimes include streak cameras, multi-frequency cross-correlation fluorimetry, and time-correlated single photon counting (TCSPC) (Lakowicz 2006; Noomnarm and Clegg 2009). Because TCSPC is the most commonly used method, we will focus on this technique. In TCSPC, a pulse of light excites a sample. A time *t* later, a fluorescence photon is detected, and the arrival time is binned. After many pulses, the binned times result in a histogram that contains the excited state lifetime convolved with the instrument response function (IRF, Appendix B). The fluorescence decay is extracted by fitting exponential decay curves to the data.

A particular difficulty in performing fluorescence lifetime experiments on intact photosynthetic samples undergoing qE is that it takes several minutes to accumulate enough counts to obtain lifetimes that have sufficiently small confidence intervals. Gilmore et al. (1995) were able to chemically pause thylakoids undergoing qE using DTT, DCMU, and methyl viologen. Similarly, Johnson and Ruban (2009) chemically “froze” chloroplasts undergoing qE by the addition of protein crosslinker glutaraldehyde. The measurement of the fluorescence lifetimes of intact leaves is complicated by the fact that turning on qE using strong light sources instead of chemical inhibitors will induce high levels of background fluorescence or saturate the detector. To address this problem, Holzwarth et al. (2009) developed a method using a rotating cuvette by which the fluorescence lifetime could be measured while qE was kept on.

Isolated, dilute chlorophyll has a fluorescence decay that is described by a single exponential decay with a time constant $\tau = \frac{1}{\sum\nolimits_i{k_i}},$ where the *k*_*i*_s are the rate constants of decay from the chlorophyll excited state (see Appendix B). Chlorophyll fluorescence lifetimes of thylakoid membranes are more complicated because of the large number of chlorophylls that can transfer energy to each other. The interpretation of these lifetimes requires a model of energy transfer in the thylakoid membrane.

Gilmore et al. (1995) fit data from thylakoids with and without qE to lifetime distributions centered at 400 ps and 2 ns. The amplitude of the 400 ps component was larger in the “qE on” state than in the “qE off” state. Because the lifetimes were conserved between the thylakoids in the two states, the lifetimes were interpreted as “puddles” of PSIIs that cannot transfer energy to one another. Within a puddle, energy transfer was assumed to occur much faster than any of the decay processes. The faster 400 ps component was attributed to PSIIs that had access to a qE site and was the first assignment of an excited state lifetime for qE. However, the measurement and model were not able to extract detailed information about the energy transfer network with qE on, such as the location(s) of quenching sites.

Understanding the energy transfer network with qE on requires a mathematical framework that incorporates that information. The equation describing the changes in excitation population on any node in the network is given by the master equation:

6 $$ \frac{{\rm d}P(t)}{{\rm d}t} = KP(t), $$

where *P*(*t*) is a vector containing the populations of each node at a time *t* and *K* is a rate matrix that contains all of the information regarding energy transfer connectivity and rates, qE and RC quenching rates, and fluorescence and ISC rates. The fluorescence decay *F*(*t*) in this formalism is simply the sum of *P*(*t*) over all nodes in the network, weighted by the rate of fluorescence at each node (Yang et al. 2003). Knowing *K* is equivalent to knowing the energy transfer network, and a full understanding of qE requires characterizing the changes in *K* between dark- and light-adapted grana membranes (see Fig. 6).

To determine *K* in grana membranes with qE on, Holzwarth and coworkers measured and fit fluorescence lifetimes on quenched and unquenched leaves with closed RCs of wild type and *npq4*, *npq1*, and L17 leaves from *A. thaliana*. A kinetic model for energy quenching in thylakoid membranes was fit to the fluorescence lifetime data using target analysis (Holzwarth et al. 2009). The kinetic model (*K*) contained the assumption that all the pigments in the grana membrane are connected, with excitation energy transfer between them occurring much faster than charge separation. The model was first fit to dark-acclimated leaves. Fitting the model with the data from light-acclimated leaves required increasing the non-radiative decay rate of the antenna compartment and including an additional compartment with a decay time of ∼400 ps. The increase in the non-radiative decay rate correlated positively with the amount of zeaxanthin, and the amplitude of the detached compartment correlated positively with the amount of PsbS. These correlations led to the proposal that there are two mechanisms of qE: one that was zeaxanthin dependent that occurred in the antenna of the PSII supercomplex, and one that was PsbS dependent that occurred by detachment of LHCII trimers from PSII. A more complex model for energy transfer in the thylakoid membrane compared to that in Gilmore et al. (1995) resulted in more detailed information about the energy transfer network.

It is still unclear what the appropriate model is for describing energy transfer in grana membranes. Recent work by van Oort et al. (2010) has suggested that the migration time of excitations in thylakoid membranes makes up ∼50 % of the average chlorophyll fluorescence lifetime. This result suggests that models that assume that energy transfer is instantaneous may not be sufficiently detailed to accurately describe energy transfer in grana membranes. Target analysis has been used to determine energy transfer models on a wide range of samples (Holzwarth 1996; van Stokkum et al. 2004). However, the fluorescence lifetime is a coarse-grained measurement, as it is a measure of the sum of all the excitation populations as a function of time. It has recently been shown that different kinetic models can fit fluorescence lifetime data equally well (Tian et al. 2013; van der Weij-de Wit et al. 2011). This means that researchers cannot necessarily differentiate between purely phenomenological models. EM and AFM measurements would allow for the determination of the relative location and orientation of proteins within the thylakoid membrane. Furthermore, the crystal structures of some individual proteins are known, which, when used with EM and AFM images, could allow for a detailed picture of the relative location of chlorophylls in the membrane. An energy transfer model that incorporates both structural information and fluorescence lifetime data would be extremely useful in identifying sites of quenching and the rates with which they quench excitation energy.

### Transient Absorption spectroscopy

Transient absorption (TA) spectroscopy is a method of probing the ultrafast dynamics intermediates involved in the photophysical mechanism of quenching. Unlike fluorescence measurements, TA can detect non-emissive species. TA measures the absorption spectrum of a sample at a fixed time after excitation (Berera et al. 2009). In TA measurements, two pulsed beams, a pump and a probe, are applied to the sample with a fixed time delay between them. The pump beam excites a portion of the chromophores in the sample. The probe beam, which is much weaker, is subsequently transmitted through the sample to measure an absorption spectrum. A difference absorption spectrum ($\Updelta A$) is calculated by subtracting the absorption spectrum of the sample without the pump pulse from the absorption spectrum when the pump pulse has excited the sample. $\Updelta A$ can then be measured as a function of wavelength *λ* and the time delay *τ* between the pump and probe pulses. The lower limit of *τ* is determined by the pulse width of the laser (for ultrafast systems this is on the order of 100 fs) and the upper limit is determined by the scanning range of the delay stage that controls the delay between the pump and probe pulses (usually around 1 ns). $\Updelta A(\lambda,\,\tau)$ is a complex quantity that may have contributions from ground state bleaching (meaning loss of absorption from the ground state), excited state absorption, stimulated emission from the excited state, and absorption from the transfer of excitation to a different molecule than the one that was initially excited. TA spectroscopy has been used to observe absorption from non-emissive intermediate states involved in qE after excitation of chlorophyll in photosynthetic proteins and thylakoid membranes.

Many groups have investigated the photophysical mechanism of qE quenching using TA spectroscopy. A large number of TA studies have been carried out examining isolated pigment-protein complexes (e.g., El-Samad et al. 2006; Müller et al. 2010; Ruban et al. 2007) as well as synthetic constructs that mimic qE in artificial systems (e.g., Berera et al. 2006; Terazono et al. 2011); a full discussion of these studies is outside the scope of this paper. Because the site of qE may not be localized on a single protein, and because the quenching properties of proteins may be altered when they are isolated from the membrane environment, correlating the results of TA experiments with qE quenching in isolated proteins is difficult. As a result, it has been necessary to study intact systems that are capable of performing qE. Thylakoid membranes are the smallest isolatable units that are capable of activating qE with light and provide a system that can be studied in solution, unlike solid-state samples such as leaves. Experiments on thylakoid membranes (Holt et al. 2005; Ma et al. 2003) have suggested that carotenoids are directly involved in the qE mechanism. Recently, a method for measuring TA during light adaptation in intact leaves was developed by the Holzwarth group (2013), which holds great promise for examining the photophysical mechanism of qE in intact photosynthetic systems.

The results of TA spectroscopy, sometimes accompanied by theoretical calculations, have led to the proposal of several different hypotheses for the photophysical mechanism of the deactivation of excited singlet chlorophyll via qE quenching: (1) the aggregation of LHCII leading to quenching by energy transfer to the lutein S_1_ state (Pascal et al. 2005; Ruban et al. 2007); (2) excitonic coupling between zeaxanthin and chlorophyll, leading to dissipation of energy via the zeaxanthin S_1_ state (Bode et al. 2009), which has also been recently observed in reconstituted proteoliposomes containing PsbS and LHCII (Wilk et al. 2013); (3) aggregation of the LHCII trimers leading to chlorophyll–chlorophyll charge-transfer state that facilities quenching (Müller et al. 2010), which has also been correlated with a red-shifted emission of chlorophyll fluorescence (Holzwarth et al. 2009); and (4) the formation of a chlorophyll–zeaxanthin charge-transfer state that quenches chlorophyll fluorescence (Ahn et al. 2008; Holt et al. 2005). These hypotheses are not mutually exclusive, but confirming or eliminating any one of them is challenging. These challenges arise from the large number of chromophores in the membrane and the lack of spectral separation between different species. For instance, chlorophyll radical cations and anions do not have distinct, sharp spectral peaks (Fujita et al. 1978), making it difficult to unambiguously prove or disprove the formation of chlorophyll radical species during qE. Carotenoid cations do have distinct spectral peaks in the wavelength range of 900–1,000 nm (Galinato et al. 2007) and have been observed to be correlated with qE in isolated thylakoid membranes (Holt et al. 2005) and isolated complexes (Ahn et al. 2008; Avenson et al. 2008). Moving forward, it seems likely that correlating the amplitudes and dynamics of TA experiments with qE in vivo will be necessary for differentiating between different qE mechanisms.

## New tools for characterizing qE in vivo

Since the first discovery of qE quenching, a great deal of information has been revealed about the triggers, components, and spectroscopic signatures associated with qE. Measurements of chloroplasts, isolated thylakoids, and isolated proteins have yielded numerous hypotheses regarding the trigger, site, and photophysical mechanisms of qE. In our view, resolving the many hypotheses that have been proposed based on isolated systems requires the development of techniques to study qE in intact living systems such as whole leaves and live algae.

**Fig. 8:**
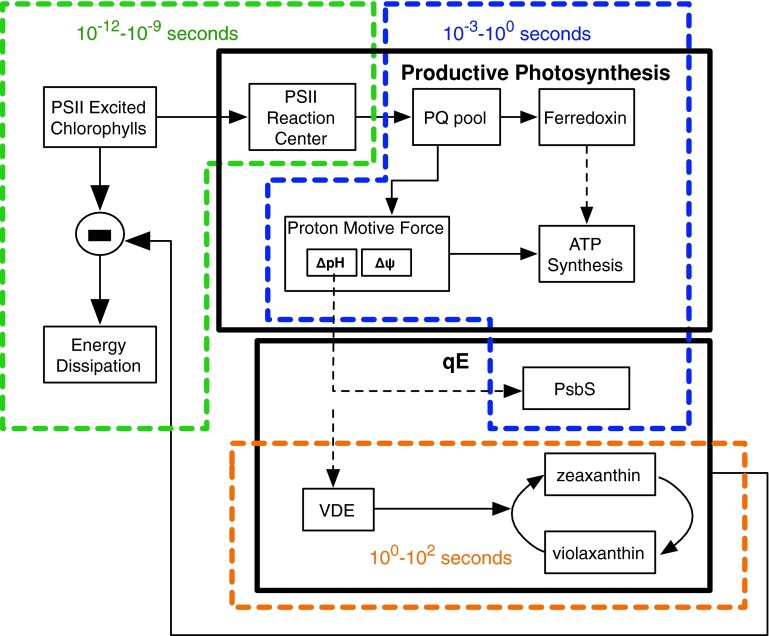
Schematic of feedback loop governing qE (*solid black rectangles*), and the broad range of timescales of processes giving rise to qE (*dashed colored rectangles*)

Because qE is a dynamic process, a full understanding requires knowledge of the timescales of constituent processes. Interpretation of results in intact systems is complicated because the events leading up to qE occur on many timescales and are affected by a large number of dynamic processes. Figure 8 illustrates the range of timescales involved in qE. In particular, the timescale of the appearance of qE quenching, as observed by fluorescence measurements, is a combination of the formation of the triggers (the lumen pH and $\Updelta\hbox{pH}$) and the timescale and set points of the membrane rearrangements (e.g., protein activations, protein aggregation) that give rise to the formation of qE. The lumen pH is itself determined by four processes: (1) water splitting at PSII, (2) proton pumping at cytochrome *b*_6_ *f*, (3) proton efflux through ATP synthesis, and (4) parsing of the proton motive force into a $\Updelta\hbox{pH}$ and a $\Updelta \psi$ component by the motion of ions across the thylakoid membrane.

The multitude of interconnected processes that give rise to a qE quenching state makes it difficult to differentiate between mechanistic hypotheses. To address this difficulty, we have developed a kinetic model of the processes in photosynthesis that give rise to qE. Our model, which is inspired by state-space models of engineering control theory analysis (Eberhard et al. 2008), calculates the lumen pH and simulates the induction and relaxation of qE in low and high light intensity (Zaks et al. 2012). The model currently consists of 24 non-linear differential equations that calculate the pH in the lumen on timescales ranging from microseconds to minutes.

**Fig. 9:**
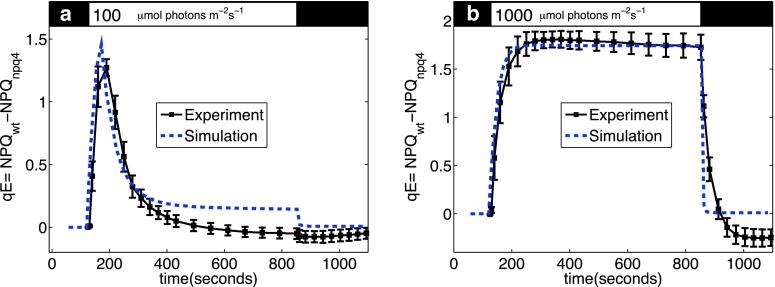
Comparison between systems model and measured qE component of NPQ in **a** low light intensity and **b** high light intensity. (adapted from Zaks et al. 2012)

We tested the effectiveness of the model by calculating chlorophyll fluorescence yields and comparing those predictions to PAM fluorescence measurements. We quantified the qE component of NPQ derived from PAM traces by subtracting the amount of NPQ developed in wild type mutant from the amount of NPQ developed in the *npq*4 mutant. Figure 9 shows a comparison between the predictions of the model and experimental measurements of rapidly reversible NPQ. The model shows good agreement with measurements of qE at 100 and 1,000 μmol photons m^−2^ s^−1^ (Zaks et al. 2012)

A benefit of using kinetic models in studying qE mechanism is that they make it possible to separate different processes giving rise to qE. For example, the timescale of qE appearance, as observed by PAM or fluorescence lifetime measurements, is affected by both the timescale of the formation of the $\Updelta\hbox{pH}$ and by the dynamics of the membrane rearrangement following qE triggering. A mathematical model such as the one we developed (Zaks et al. 2012) provides a framework for testing hypotheses of many mechanisms relating to qE. For instance, it is not clear whether the pH-sensing components of the membrane have a fixed p*K*_a_, as assumed in Takizawa et al. (2007), or have a variable p*K*_a_, as proposed in Johnson and Ruban (2011) and Johnson et al. (2012). It is possible to quantify these two hypotheses using mathematical expressions, then integrate both expressions into the model and compare the predictions of either hypothesis. Additionally, as mathematical models of individual components are developed and refined, these models could be integrated in a modular fashion into the framework of a systems model to test the implications of a detailed understanding on the behavior of the thylakoid system as a whole. To aid this effort, we have made the documented MATLAB code of our model available (Zaks). We have also created a GUI for our model that facilitates the exploration of the model by researchers from a broad range of backgrounds (Zaks 2012).

A challenge associated with experimentally testing the predictions of kinetic models is that methods for measuring qE typically measure either slow biochemical changes (sec to min timescale, which can be characterized using PAM) or the fast dynamics in the light-harvesting antenna (fs to ns timescale, by measuring fluorescence lifetimes or TA) in dark- or light-acclimated samples. Understanding how the triggers/components of qE act in concert to activate quenching requires a technique that bridges both slow and fast timescales. The photophysical mechanisms and sites involved in qE are intimately tied to the biochemical and physical changes that occur to activate these mechanisms.

**Fig. 10:**
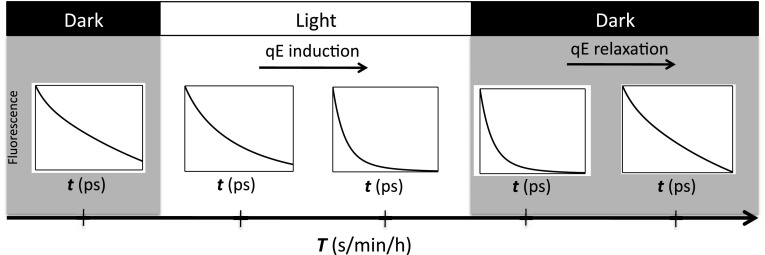
Schematic of “fluorescence lifetime snapshots” measurements. The technique tracks changes on both the *T* timescale (sec to hours) as well as in the *t* timescale (ps to ns). qE triggering and the thylakoid membrane rearrangement occur on the *T* timescale. Quenching of chlorophyll fluorescence occurs on the *t* timescale and contains information about the membrane configuration

To fill this gap in techniques for measuring qE, we have developed a technique for measuring the changing fluorescence lifetime as qE turns on in plants and algae, which we call “fluorescence lifetime snapshots” (Fig. 10) (Amarnath et al. 2012). It is a two-dimensional (2D) technique with one time axis being the fluorescence decay time and the second being the adaptation/relaxation timescale. The technique has so far been used to measure the changes in fluorescence lifetimes in live cells of *Chlamydomonas reinhardtii* during the transition from a dark- to a light-acclimated state, and back to a dark-acclimated state. The data from the measurement on algae were globally fit to three exponential decays. This result suggested that the three lifetimes could be treated as separate pools of PSII that cannot transfer between each other. Two of the populations had lifetimes of 65 and 305 ps, with the third having a lifetime of 1 ns. The amplitudes of the two shorter lifetimes increased during the light treatment and decreased in the ensuing darkness. In addition, these amplitudes substantially decreased when the pH gradient was dissipated using nigericin. The amplitudes associated with the 65 and 305 ps lifetime components exhibited different dynamics during qE induction and relaxation, which led us to suggest that there are two different mechanisms associated with qE in *C. reinhardtii*. This technique correlates the *T* axis, which describes the timescales of qE triggering, with the *t* axis, which probes changes in the membrane and photophysical mechanism of qE.

As discussed in the “Fluorescence lifetimes” section and Appendix B, the insight from fitting fluorescence lifetimes to multiple exponential decays is limited. Using the fluorescence lifetime snapshot measurements to differentiate between different hypotheses for qE mechanisms requires fitting the fluorescence lifetimes to a detailed mechanistic model of energy transfer. Because different energy transfer models are able to fit fluorescence lifetime data well (van der Weij-de Wit et al. 2011), much theoretical and experimental progress remains to be made in developing accurate models of energy transfer in PSII. We are optimistic that future developments in this area will enable the interpretation of fluorescence lifetime snapshots in the context of a mechanistic model for qE.

## Concluding remarks

Looking forward, much progress in the development of experimental techniques and theoretical models will be needed before the site(s) and mechanism(s) of qE are identified and the triggering processes and ensuing membrane changes are characterized. Obtaining unambiguous answers is particularly challenging because the pigments and proteins involved in qE are found inside of a lipid membrane, are buried within a cell, are highly dependent on interactions with their local environment, and undergo changes on a wide range of timescales. To address these challenges, it will be necessary to develop experimental techniques with high spatial, spectral, and temporal resolution that can probe dense, heterogeneous membrane-based systems. It is also becoming possible, and will likely be necessary, to develop mathematical models that take advantage of increasingly powerful computing power to encompass the true complexity of qE. It will be important that these models be capable of making falsifiable predictions that enable differentiation between different mechanisms of qE. Such developments should provide valuable, as understanding a detailed mechanism of qE would profoundly extend our understanding of the regulation of biological energy transduction and will likely provide useful design principles for the regulation of light harvesting in fluctuating light conditions.

## Appendix A: Pulse amplitude modulated fluorescence

A typical PAM trace of a wild type leaf of *A. thaliana* is shown in Fig. 2. At the beginning of the PAM trace, the actinic light source is off. Then, a 1-s saturating flash is applied, and the maximum fluorescence measured during the flash is called *F*_m_. Using a simplified definition of chlorophyll quantum yield described in Ahn et al. (2009) and Hendrickson et al. (2005), we can write *F*_m_ as

7 $$ F_{\rm m} \propto \varPhi_{F,F_{\rm m}} = \frac{k_{\rm F}}{k_{\rm F} + k_{\rm IC} + k_{\rm ISC}}, $$

where $\varPhi_{F,F_{\rm m}}$ is the fluorescence quantum yield during the measurement of *F*_m_ and *k*_F_, *k*_IC_, and *k*_ISC_ are the rate constants of decay for fluorescence, internal conversion, and intersystem crossing, respectively (Ahn et al. 2009). There, rate constant for photochemistry at the RC in the denominator is equal to 0 because the saturating pulse closes all RCs and temporarily blocks photochemistry. After the actinic light, bar at top of plot, is turned on, a saturating pulse is applied every minute. The actinic light remains on for 10 min, followed by darkness for 10 min. The maximum fluorescence yield during each of these pulses is called $F_{\rm m}^{\prime},$

8 $$ F_{\rm m}^{\prime} \propto \varPhi_{F,F_{\rm m}^{\prime}} = \frac{k_{\rm F}}{k_{\rm NPQ}(T) + k_{\rm F} + k_{\rm IC} + k_{\rm ISC}}, $$

where *k*_NPQ_ is the rate constant for dissipation by NPQ. Note that $F_{\rm m}^{\prime}$ is time dependent because *k*_NPQ_ is time dependent.

There are many ways to extract the dynamics and the amplitude of the qE component of quenching from a PAM trace. One way is by measuring the fluorescence after qE has relaxed (with other components of NPQ such as qI and qT still intact); called $F_{\rm m}^{\prime\prime},$ it is possible to estimate the amount of qE (Demmig and Winter 1988):

9 $$ \hbox{qE} = \frac{F_{\rm m}^{\prime\prime}-F_{\rm m}^{\prime}}{F_{\rm m}^{\prime\prime}}. $$

This qE parameter can be used to see what components or chemicals affect the amplitude of qE (Johnson and Ruban 2011). Additionally, it is possible to estimate the quantum yield of qE, $\varPhi_{\rm qE}.$ by additionally measuring *F*_S_, the fluorescence yield, immediately before a saturating pulse is applied.

10 $$ \varPhi_{\rm qE} = \frac{F_{\rm m}^{\prime\prime}-F_{\rm m}^{\prime}}{F_{\rm m}^{\prime\prime}} \frac{F_{\rm S}}{F_{\rm m}^{\prime}} $$

where *F*_S_ is the fluorescence of the PAM trace right before a saturating pulse is applied (Ahn et al. 2009).

## Appendix B: Time-correlated single photon counting

In this section, we describe the basic principles of TCSPC. A short pulse of light is used to excite a fluorophore such as chlorophyll. Free chlorophyll in solution in the excited state can relax back to the ground state via fluorescence, IC, or ISC. The rate constant for each decay process does not depend on the time that the chlorophyll has been in the excited state. A photon of fluorescence is detected at time $t + \Updelta t$ after excitation. The experiment is repeated many times, with many photons of fluorescence observed and binned (with bin width equal to $\Updelta t$) to make a histogram. This histogram has a shape defined by the probability *P*(*t*) that the chlorophyll molecule is in the excited state at time $t=M\Updelta t.$ If, after a $\Updelta t$ timestep, the probability that the chlorophyll molecule is still in the excited state is $1 - (k_{\rm F} + k_{\rm IC} + k_{\rm ISC})\Updelta t,$ it follows that

11 $$ P(t) = \left(1-\left(k_{\rm F} + k_{\rm IC} + k_{\rm ISC}\right)\frac{t}{M}\right)^M, $$

In the limit that $\Updelta t$ goes to 0, or *M* goes to infinity,

12 $$ P(t) = \lim_{M\to\infty} \left(1-\left(k_{\rm F} + k_{\rm IC} + k_{\rm ISC}\right)\frac{t}{M}\right)^M = \exp \left( \frac{-t}{k_{\rm F} + k_{\rm IC} + k_{\rm ISC}} \right). $$

The form of the decay of the population of chlorophyll excited states goes as an exponential with a time constant $\tau = \frac{1}{k_{\rm F} + k_{\rm IC} + k_{\rm ISC}}.$

The width of the light pulse and the response time of the instrument are convolved with the fluorescence decay of the sample. To extract the decay, *F*(*t*) (analogous to *P*(*t*) above), requires a reconvolution fit of the data *I*(*t*),

13 $$ I(t) = \int\limits_{-\infty}^t {\rm IRF}(t^{\prime}) \sum\limits_{i}^n A_i {\rm e}^{\frac{-t-t^{\prime}}{\tau_i}}, $$

where IRF is the instrument response function. It is often assumed that $F(t) = \sum\nolimits_{i}^n A_i {\rm e}^{\frac{-t}{\tau_i}},$ where *n* is the number of exponentials, *A*_*i*_ are the amplitudes, and *τ*_*i*_ are the lifetimes. *A*_*i*_ and *τ*_*i*_ are fit to the data using a criterion such as least-squares or maximum likelihood (Lakowicz 2006). Measurements of the fluorescence lifetime of the chlorophyll in the thylakoid membrane exhibit more complicated decay dynamics (see Fluorescence lifetimes section).
